# Towards first-principles molecular design of liquid crystal-based chemoresponsive systems

**DOI:** 10.1038/ncomms13338

**Published:** 2016-11-02

**Authors:** Luke T. Roling, Jessica Scaranto, Jeffrey A. Herron, Huaizhe Yu, Sangwook Choi, Nicholas L. Abbott, Manos Mavrikakis

**Affiliations:** 1 Department of Chemical and Biological Engineering, University of Wisconsin-Madison, 1415 Engineering Drive, Madison, Wisconsin 53706-1607, USA

## Abstract

Nematic liquid crystals make promising chemoresponsive systems, but their development is currently limited by extensive experimental screening. Here we report a computational model to understand and predict orientational changes of surface-anchored nematic liquid crystals in response to chemical stimuli. In particular, we use first-principles calculations to evaluate the binding energies of benzonitrile, a model for 4′-pentyl-4-biphenylcarbonitrile, and dimethyl methylphosphonate to metal cation models representing the substrate chemical sensing surface. We find a correlation between these quantities and the experimental response time useful for predicting the response time of cation–liquid crystal combinations. Consideration of charge donation from chemical species in the surface environment is critical for obtaining agreement between theory and experiment. Our model may be extended to the design of improved chemoresponsive liquid crystals for selectively detecting other chemicals of practical interest by choosing appropriate combinations of metal cations with liquid crystals of suitable molecular structure.

## Abstract (web summary)

Nematic liquid crystals have potential as sensors for various molecules. Here, the authors present a computational chemistry model for describing the detection of a warfare agent by liquid crystals, opening the door for the atomic-scale design of sensitive and selective chemoresponsive systems.

## Graphical abstract

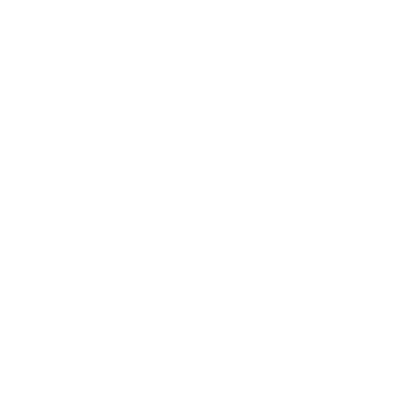

An emerging trend in materials design is the use of computational chemistry as a driver for materials discovery, with notable successes in the design of heterogeneous catalysts^1^ and batteries^2^, among others. Investigations of the anchoring of liquid crystals (LCs) to chemically functionalized surfaces and their subsequent orientational transition upon exposure to targeted analyte molecules are leading to the development and improvement of chemical and biological sensors^3^,^4^,^5^,^6^,^7^,^8^, although these innovations have required slow, laborious experiments not yet informed by computational materials discovery. In these sensors, the orientational ordering of LCs depends primarily on the chemical functionality of the surfaces used to support the LCs^9^,^10^. A previous study demonstrated that the mesogen 4′-pentyl-4-biphenylcarbonitrile (5CB), which forms a nematic LC phase, can assume a parallel or tilted orientation relative to surfaces decorated with certain metal cations and a homeotropic orientation (perpendicular to the surface) relative to surfaces decorated with other cations^11^. Homeotropic anchoring of 5CB was inferred on the basis of FTIR spectroscopy to occur through interactions of the nitrile group of 5CB with the cation. The subsequent exposure of the supported LC film to dimethyl methylphosphonate (DMMP), a common simulant of sarin nerve gas, can lead to a change in the orientation of the LC, depending on the metal cation identity. Evidence of the molecular interactions underlying the responses was obtained by using infrared spectroscopy for the cations Cu(II) and Ni(II) with 4′-octyl-4-biphenylcarbonitrile (8CB) and DMMP^12^. These results suggest that a more detailed understanding of the interactions between metal cations and 5CB, and the subsequent displacement of 5CB by DMMP, would provide useful fundamental information for developing a universal approach capable of predicting possible orientational transitions of LCs, which are initially anchored on surfaces containing specific metal cations, upon exposure to a specified analyte.

Here, we report the main results of quantum-chemical studies of the interaction of metal cations with benzonitrile (PhCN), serving as a surrogate molecule for 5CB, and DMMP. The displacement of PhCN by DMMP is predicted on the basis of the thermochemical binding energies (BEs) between each of the PhCN and DMMP molecules and the metal ion. We utilize an iterative approach in which cycles of experiments and computation create a feedback loop to unify model predictions with experimental detection properties. The results of our calculations establish a robust theoretical model for the prediction of LC responses to selected analytes of interest, and open the possibility for the accelerated design of improved chemoresponsive materials through a combined theoretical, experimental and LC-synthetic approach.

## Results

### Minimum energy structures and binding energies

Key parameters of the energy-optimized structures of PhCN and DMMP are given in Supplementary Tables 1 and 2, Supplementary Fig. 1 and the Supplementary Discussion. We observed good agreement between our computed values and previously obtained experimental^13^ and computed^14^ values.

**Figure 1:**
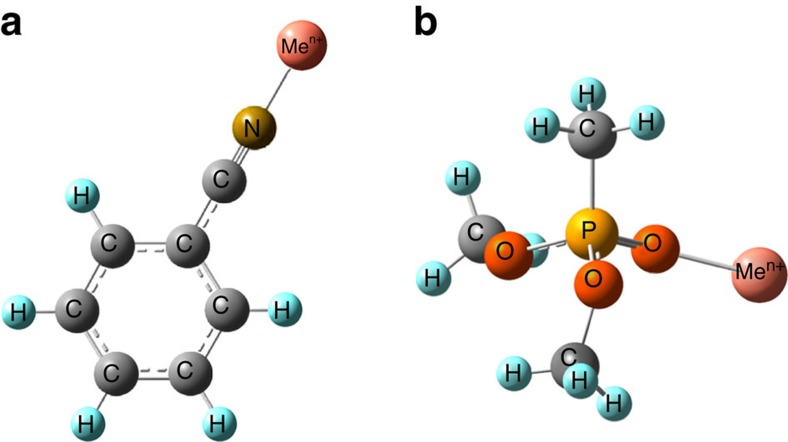
Energy-optimized adsorption geometries. Shown are the minimum energy structures of metal cations (Me^n+^) bound to (**a**) benzonitrile (PhCN) and (**b**) dimethyl methylphosphonate (DMMP). Structural details for all cations studied are given in Supplementary Tables 3 and 4.

In our first computational cycle, we calculated the binding energies of PhCN or DMMP to metal cations using the formal charge on the metal cations as determined from the metal salt precursor used in the preparation of the substrate surface (the `formal charge' approach). For example, a substrate surface created by using Cu(ClO_4_)_2_ as a precursor salt was modelled using a Cu^2+^ cation. The main structural parameters of the Me^n+^-PhCN and Me^n+^-DMMP complexes are provided in Supplementary Table 3 and the Supplementary Discussion. Representative structures of typical Me^n+^-PhCN and Me^n+^-DMMP complexes are shown in Fig. 1. We note that our subsequent calculations will involve cations in varying oxidation states. To avoid confusion, all cations described in this manuscript will be listed using their formal charges from the experimental perchlorate salt precursor (unless specifically noted otherwise).

The calculated binding energies (BE_PhCN_ and BE_DMMP_) and final charges on the metal cations (*q*_f_) are shown in Table 1. The differences between BE_PhCN_ and BE_DMMP_ are also provided, and are used to predict the displacement of 5CB by DMMP when DMMP binds more strongly by at least 0.20 eV (see Methods section). Using this model, agreement between previous experiments^11^ and theoretical predictions of displacement was found in only one case of the ten metal cations considered (displacement of 5CB from La^3+^). Importantly, we also calculated substantial values of BE_PhCN_ for Ag^+^ (−2.21 eV) and Na^+^ (−1.41 eV), which do not exhibit homeotropic anchoring of 5CB experimentally. These significant binding energies suggest that homeotropic anchoring should occur, as they are much larger in magnitude than the threshold value adopted for weak interactions between the LC and metal cations (−0.20 eV). This suggested that our formal charge model is overpredicting the binding energies of liquid crystals to metal cations, which is supported by the other very large binding energies shown in Table 1.

The poor agreement between the first cycle of calculations and the experimental observations motivated the use of an alternative approach for calculating binding energies. Specifically, molecular details of the LC–substrate interface led us to hypothesize that several species (for example, solvent or counterion) may donate electron density to the metal cation (see below for details). Accordingly, we performed a second cycle of calculations in which the charge on the metal cation was reduced from the formal charge by one unit (the `reduced charge' approach; structural results given in Supplementary Table 4). The binding energies (Table 2) calculated with this model are substantially weaker than the formal charge values due to the reduced electron affinities of the cations in their reduced charge states. We found that all cations that bind to PhCN with a binding energy of at least −1.9 eV (that is, the strength of the reduced-charge Cd^+^ binding) exhibit homeotropic anchoring experimentally. PhCN binds more weakly to Ag^0^ and Na^0^ (reduced charges) than the adopted −0.20 eV threshold (as opposed to stronger than −1.0 eV using the formal charge approach), suggesting that only very weak interactions occur between these metals and PhCN, and as a result, the LC assumes a parallel (non-homeotropic) orientation, in agreement with experimental observations. The difference between BE_DMMP_ and BE_PhCN_ on Co^+^ (reduced charge) is very small (−0.03 eV) and less negative than the cutoff value of −0.20 eV we define for displacement to occur; our prediction of no displacement on Co^+^ agrees with the experimental observation. In total, we found agreement between reduced charge model calculations and experiment regarding displacement events in seven of the ten cases studied.

### Experimental evaluation of response time

These theoretical results motivated an additional cycle of experiments. The original experimental system on which this study was based^11^ focused on whether or not the liquid crystals responded to DMMP and not the dynamics of those responses. Since our theoretical model predicts a magnitude of displacement energy to which dynamics might be related, we performed a new subset of experiments with an improved methodology that reduced the role of interactions other than cation-mesogen bonds in the response of the LC (see Methods for a description of differences between the old and improved experimental protocols). We used this new experimental system to explore the relationship of the calculated displacement energy and the experimentally measured response time. A sample experimental response for 5CB anchored to Al^3+^ is provided in Supplementary Fig. 2.

**Figure 2:**
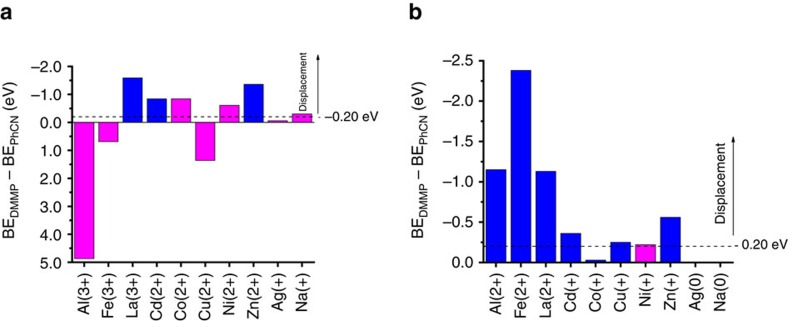
Comparison of displacement using the formal charge approach and the reduced charge approach. Shown are the calculated displacement energies (BE_DMMP_–BE_PhCN_) for displacement of benzonitrile (PhCN) by dimethyl methylphosphonate (DMMP) using (**a**) the formal charge approach, and (**b**) the reduced charge approach. Displacement is predicted to occur when the displacement energy is stronger (more negative) than −0.20 eV. Blue bars represent agreement between theoretical predictions and experimental observations, whereas magenta bars show disagreement. Ag^0^ and Na^0^ are not predicted to exhibit homeotropic anchoring (in agreement with experiments), so no colour bar is shown for those entries.

This new cycle of experiments found that Zn^2+^ exhibited a response to DMMP, and that Cd^2+^ exhibited a weak but variable response (consistent with its small calculated displacement energy). These new experimental results agree with the computational predictions of the reduced charge model. Furthermore, the model predicted the highest displacement energy for Fe^3+^ (charge from metal salt precursor), and Fe^3+^ was found to exhibit the fastest response in the new experiments. In total, six cations were found to respond to DMMP exposure (increased from four, previously). As before, Ni^2+^ and Co^2+^ did not undergo a transition from homeotropic anchoring, and Ag^+^ and Na^+^ did not exhibit homeotropic anchoring. A graphical comparison of the predicted displacement energies and agreement between the calculated displacement energies (both formal and reduced charge approaches) and the new experimental cycle is shown in Fig. 2. As shown in the figure, agreement between the new experiments and the reduced charge calculations was found in nine of ten cases; the remaining point of disagreement (Ni) corresponds to a displacement energy (−0.22 eV) very close to our cutoff value of −0.20 eV.

**Figure 3:**
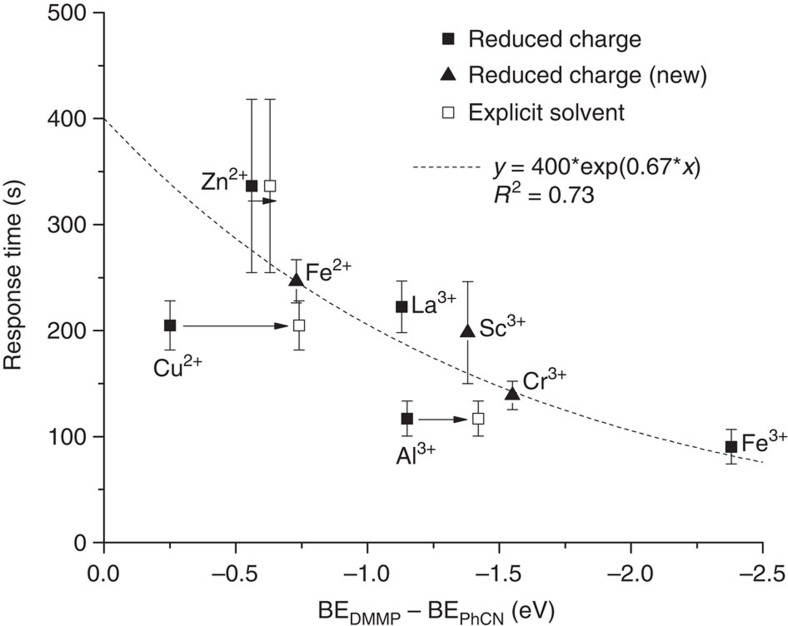
Computed binding energies predict experimental displacement time. Shown is the experimental response time of 5CB anchored to various metal salts upon exposure to dimethyl methylphosphonate (DMMP) as a function of calculated displacement energy. The response time was defined as the time required to reach 80% normalized light intensity. Filled squares represent values for metal cations in the original data set of reduced charge calculations and corresponding experiments. The best-fit curve was calculated from these original data points, with the exception of the Cu outlier. Filled triangles represent new (Cr^3+^, Sc^3+^ and Fe^2+^) cations with response times predicted by the reduced charge model and evaluated experimentally. The hollow data points represent data calculated from a solvent-explicit model, as described in the text. Experimental error bars are drawn one standard deviation from the mean. All charge designations shown in the figure correspond to the metal salt precursor charges from experiments.

The response times for the cations that demonstrated an anchoring transition are plotted as an exponential function of the calculated displacement energies in Fig. 3 (Supplementary Discussion). A reasonable correlation between these quantities is found (*R*^2^=0.73), with the exception of Cu^2+^, which we address more fully in the next section, and Cd^2+^, which was omitted due to its weak and variable response. This result demonstrates that the difference between BE_DMMP_ and BE_PhCN_, despite being a purely thermodynamic quantity (no activation energy barriers for the displacement event were calculated), may be a reasonable predictor of the response time of LC films to DMMP exposure.

To evaluate the predictive capability of our model, we calculated the binding energies of three additional metal cations (formal charges: Sc^3+^, Cr^3+^, Fe^2+^) to PhCN and DMMP using the reduced charge model, and measured their experimental response times. We found that all three systems responded to DMMP and their response times were predicted well by the reduced charge model binding energies to the respective metal cations (Fig. 3). These results validate the predictive capability of the model, and demonstrate its utility in the future design of improved chemoresponsive materials on the basis of calculated binding energies.

### Effect of charge transfer from solvent

We performed additional calculations to improve our understanding of why the reduced charge approach leads to better agreement with experiment relative to the formal charge approach, choosing Cu^2+^ for further study since it was an outlier in the data set shown in Fig. 3. In the experiments, an ethanolic solution of each metal salt (in this case, Cu^2+^) was spin-coated onto a substrate to prepare the metal surface. The solvent was then evaporated, but we postulate that: (i) some residual ethanol (EtOH) molecules might remain bound to the metal cation as a result of a possible strong interaction between the cation and EtOH in solution, and (ii) that the charge of the solvated metal cation is therefore different from its formal charge due to donation of electron density from the EtOH solvent to the cation. To test this hypothesis, we calculated the differential binding energy of EtOH and optimized cation charge resulting from the relaxation of a Cu^2+^ cation (formal charge) in the presence of *n* EtOH molecules (*n*=1–4). These calculations showed that: (i) the cation charge decreases with increasing *n*, and (ii) EtOH molecules were bound progressively more weakly along with this decrease in charge as *n* increased (Supplementary Fig. 3; Supplementary Discussion).

To further investigate this phenomenon, we computed BE_PhCN_ and BE_DMMP_ to Cu^2+^ in the presence of one and two EtOH molecules (Supplementary Discussion). We found that calculations in the presence of two EtOH molecules particularly resembled the reduced charge model, obtaining BE_PhCN_=−2.99 eV and BE_DMMP_=−3.73 eV. These values are within 1 eV of those calculated with the reduced charge approach (−2.56 eV, −2.81 eV), demonstrating reasonable qualitative agreement between this larger model containing Cu^2+^ bound to two EtOH molecules and the reduced charge model. Moreover, the displacement energy calculated in the complex containing two EtOH molecules (−0.74 eV) indicates that displacement of PhCN by DMMP will occur, in agreement with the reduced charge model and with the experiments. This displacement energy on Cu^2+^ calculated in the presence of two EtOH was added to Fig. 3 as a hollow square. We also note that the final charge on the Cu^2+^ cation (0.68) in the presence of two EtOH resembles the (1+) charge assigned to it in the reduced charge model. We performed the same calculations with two EtOH for Zn^2+^ and Al^3+^ and found that shifts in BE_DMMP_–BE_PhCN_ also existed for these (by −0.07 eV and −0.27 eV, respectively), though these were smaller in magnitude than the shift in BE_DMMP_–BE_PhCN_ for Cu^2+^ (−0.49 eV), which was a particular outlier from the calculated curve in Fig. 3. Two of the three explicit-solvent points (Cu^2+^ and Al^3+^) were substantially closer to the correlation established by the reduced-charge data points, while the Zn^2+^ value was only slightly further from the correlation; this demonstrates that our model may continue to be improved (albeit at greater computational expense) by explicit inclusion of solvent molecules in future computational cycles.

**Figure 4:**
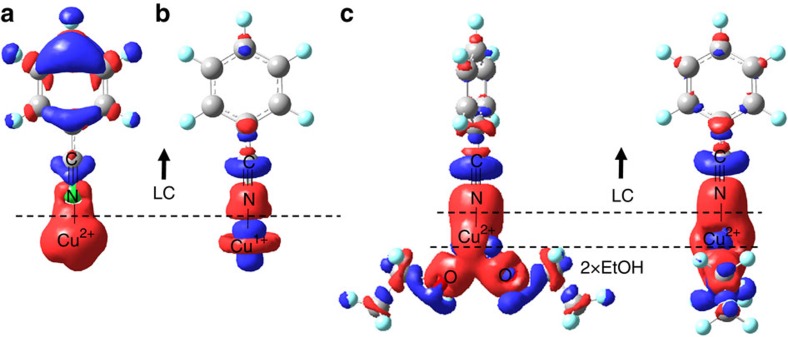
Charge transfer diagrams. Shown are calculated charge density difference plots for the interaction between a Cu cation and benzonitrile (PhCN), using (**a**) the formal charge approach, (**b**) the reduced charge approach, and (**c**) the formal charge approach incorporating two ethanol (EtOH) solvent molecules (two views of the same complex are provided for clarity). EtOH donates electron density to Cu^2+^, lowering its effective charge. Electronic charge density depletion from the aromatic ring is significantly reduced in the presence of EtOH, as in the case of the reduced charge model shown in (**b**). Red represents electron charge accumulation and blue electron charge depletion; an isovalue of 0.005 e Å^−3^ was used to construct the surfaces.

We constructed charge density difference plots, shown in Fig. 4, to illustrate the effects of charge donation from EtOH molecules in the Cu^2+^ system. When using the formal charge approach, as in Fig. 4a, significant electronic charge depletion is observed on the aromatic ring of PhCN to form the Cu-N bond. This substantial donation of electron density from PhCN to Cu^2+^ explains the exceptionally negative (i.e., strong) BE_PhCN_ calculated when using this approach. In contrast, when using the reduced charge approach as shown in Fig. 4b, the Cu–N bond is formed primarily by donation of electrons only from the C–N bond; minimal impact is observed on the aromatic ring's electron density, explaining the weaker binding energy when using the reduced charge approach.

When performing calculations using the formal charge on the metal cation in addition to two EtOH molecules, as shown in Fig. 4c, we observe that electron density is not substantially drawn from the aromatic ring, similar to the reduced charge case. Electron density is instead donated from the EtOH molecules, in addition to the C–N bond, leading to a strong qualitative agreement with the reduced charge approach results (considering only the effect on the aromatic ring). We conclude that the reduced charge model, which shows good agreement with the experimental observations, represents reasonably well the interaction between the formal charge metal cations and PhCN or DMMP in the presence of two EtOH molecules, which may be more representative of the true physical environment of the metal cation. The ability to utilize the reduced charge model in place of the explicit-solvent formal charge model is important for reducing the computational cost of future calculations, due to the substantially decreased system size treated in the reduced charge model. However, we observe that the explicit inclusion of such solvent effects in future computational cycles could offer improved accuracy by fine-tuning the charge reduction on metal cations interacting with LCs and analytes; in particular, systems for which disagreement is observed between theory and experiment could be further studied by an explicit solvent model rather than a coarse-grained approximation of charge reduction by reducing by one integer unit, as we have done for Cu^2+^.

We note briefly that this charge transfer can also be affected by the presence of other species (instead of EtOH) present in the environment of the sensing surface, including water or the counterions in the salt precursor used to prepare the surface. We do not attempt to quantify the effects of charge transfer from other species in this work, but merely note that their effects on cation charge could be qualitatively similar to the effect of EtOH (Supplementary Discussion).

### First-principles design of selective chemoresponsive systems

The present computational model was developed in the context of predicting the orientational transitions of 5CB-based LCs, as represented by PhCN, upon exposure to DMMP. However, the principles behind this model are general and may be extended to guide the first-principles design of LC-based sensors for new classes of molecules/analytes. In particular, we identify three primary degrees of freedom that may be explored in future models: the identity of the analyte, the identity of the metal cation, and the molecular structure of the liquid crystal.

First, the choice of analyte is not limited to DMMP, but could in principle include any molecule binding to metal cations with sufficient strength to displace a LC. The selection of analytes will be dictated by demand for highly sensitive and selective detection in a range of applications, including (but not limited to) medical, security and generic industrial safety applications.

Second, as illustrated in this study, the choice of metal cation strongly influences the detection capabilities of these LCs. The metal cations explored computationally thus far only represent a subset of those available for experimental applications. By performing calculations on a greater set of metal cations in a range of commercially available oxidation states, computational screening can identify combinations of LCs and metal cations with improved sensitivity toward detection of specific analytes of interest. In particular, computations will seek to identify metal cations that bind particularly strongly to an analyte of interest, so that the analyte might displace a weaker-binding LC from the metal cation binding site.

Third, the methods described in this study may be applied to the selective detection of chemical analytes by comparing their relative binding energies to individual metal cations (in addition to the binding energy of the LC to the metal cation). To briefly explore this possibility, we note that water (in the form of ambient humidity) is ubiquitous in most sensing applications and has the potential to generate a ‘false-alarm' with a LC-based sensor. We evaluated the binding energies of water to all the metal cations considered in this study (Supplementary Table 5). For those cations exhibiting homeotropic anchoring experimentally (that is, excluding Na^0^ and Ag^0^), we found that water binds much more weakly than PhCN (by at least 0.45 eV) and DMMP (by at least 0.80 eV). This suggests that water should not displace PhCN and would be non-competitive with DMMP detection. We performed an additional set of detection experiments by exposing supported films of 5CB to ambient humidity (Supplementary Fig. 4), which showed that the liquid crystal orientation was indeed unaffected by the presence of ambient humidity upon extended exposure for one hour, in complete agreement with our model predictions. The possibility of selective detection suggests the future design of systems with liquid crystals and metal cations specifically chosen to yield a fast, selective response to a targeted chemical.

Fourth, the binding properties of the LCs themselves can be adjusted through modification of the functional groups through which they bind to the metal cations, as well as general modification of their detailed larger molecular structure. In particular, replacement of the nitrile termination in 5CB/PhCN with other functional groups can tailor the LC-metal cation interaction to the optimal strength for specific analyte detection. Ideal LCs will bind to metal cations more weakly than the 5CB/PhCN studied in this work, so that displacement of the liquid crystal will be more favourable thermodynamically. However, LCs must still bind sufficiently strongly to enable homeotropic anchoring to the metal cations, so binding energy optimization must be carefully considered. Quantum chemical calculations can definitely inform and guide the organic synthesis of the respective promising LC molecules.

Finally, our calculations have shown that the exact method of synthesizing specific chemoresponsive materials, including solvents used in the synthetic protocol, might be selectively adjusted to fine-tune the effective charge on the metal cation, thereby providing a useful secondary degree of freedom worth considering in future design of LC-based sensors.

## Discussion

We successfully developed a first-principles approach to predict the displacement of 5CB, represented by benzonitrile (PhCN), by dimethyl methylphosphonate (DMMP) on the basis of calculated thermochemical binding energies utilizing integrated cycles of theory and experiment. We found that decreasing the initial metal cation charge by one unit relative to that in the metal salt used to anchor the LC molecules leads to a better prediction of LC displacement by DMMP. The better performance achieved by decreasing the initial charge of the metal cations was rationalized by the effect of electronic charge transfer from residual EtOH solvent used in the experimental preparation of the surfaces exposing the metal cations. Moreover, we elucidated a correlation between the predicted displacement energy of DMMP by PhCN and the experimentally measured orientational response time of 5CB to DMMP that can be used to predict the response time of new chemoresponsive systems. Our calculations additionally predict that these should be no chemo-response with respect to water, as water binds much more weakly than do both DMMP and PhCN, which explains the lack of experimental response of the supported LC to ambient humidity. Finally, although this model was developed in the context of DMMP detection by 5CB, the principles and methods established in this study are general and may be applied for developing sensors for the selective detection of a broad range of analytes by a variety of liquid crystals, which could open opportunities towards a molecular-level design of novel sensors based on first-principles methods.

## Methods

### Computational

We initially considered periodic slab models to describe the interactions between the metal cations and PhCN/DMMP. In particular, we attempted planewave DFT calculations on extended surfaces of pure metals (for example, (111) or (100) facets of fcc metals), but found poor agreement between these models and experimental results. We also considered models in which the metal ions were charged. We finally considered surface models of bulk metal perchlorate salts, but found that these models were too complicated due to the varying stoichiometry and crystal structures, along with the very high computational costs associated with such models, rendering them impractical for our modelling purposes. We therefore developed the simple, approximate but more straightforward modelling approach described in the following sections, in which the surface is replaced by an appropriately charged metal cation.

Calculations were performed with *Gaussian 09* (ref. 15) using the CBS-QB3 Complete Basis Set method, a highly accurate composite method available for atomic numbers up to 36 (refs 16, 17). In the CBS-QB3 method, the energy-optimized structures and the zero-point energy (ZPE) were computed at the B3LYP/CBSB7 level of theory. Calculations involving metals with atomic number higher than 36 (that is, La, Cd and Ag), for which the CBS-QB3 method was not available, were performed at the B3LYP level^18^ using the compact effective potentials CEP-121G basis set^19^,^20^. The Counterpoise method^21^,^22^ was used to correct the BEs for the basis set superposition error (BSSE) for those metals computed at the B3LYP/CEP-121G level of theory.

The binding energies of PhCN and DMMP (BE_PhCN_ and BE_DMMP_) to metal cations were calculated as the difference between the total energy of the complex of the metal and the molecule (*E*_complex_) and the sum of the total energies of the isolated Me^n+^ (*E*_Me_) and the isolated molecule (*E*_molecule_):


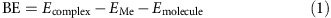


A negative value of BE indicates that the formation of the complex between the metal cation and the molecule is a thermodynamically favourable process, whereas a positive BE indicates an unfavourable process.

Our energy optimization calculations for the Me^n+^-PhCN and Me^n+^-DMMP complexes consider only binding of the metal cations to the N atom of the nitrile group (for PhCN) or the phosphoryl O atom (for DMMP). This treatment is consistent with FTIR spectroscopy that identified these as the primary functional groups through which binding occurs^11^,^12^. Binding of metal cations to other functional groups/atoms on those molecules was briefly investigated and found to be much weaker, and is therefore neglected in the following analyses.

Considering the accuracies of both adopted methods (CBS and B3LYP), which are typically accurate for calculating absolute quantities (in this case, binding energies) to within 0.1–0.2 eV, and after comparing our model predictions with available experimental data, we assume that displacement of PhCN by DMMP will take place when, for a particular metal cation, BE_DMMP_ is at least 0.20 eV stronger (more negative) than the BE_LC_, that is: BE_DMMP_–BE_PhCN_<−0.20 eV. We expect no displacement to take place if this difference is more positive than −0.20 eV. We note that the cutoff energy is a parameter that accounts for the uncertainty associated with the calculated binding energies as well as additional sources of error arising from complex environmental factors not considered by the model. For example, a gas-phase reference state of isolated molecules is not physically representative of the liquid-phase environment of these molecules; relatively weak intermolecular interactions (relative to binding of 5CB/DMMP to the metal cation) such as those between 5CB molecules and between DMMP and 5CB are implicitly accounted for in this cutoff parameter. Entropic corrections to the free energy of displacement are also not explicitly accounted for, and represent an additional source of error.

Furthermore, in order for homeotropic anchoring to occur, we require a threshold (negative) binding energy between the metal cation and the PhCN molecule. In particular, again considering the typical computational accuracy and the effects of other factors neglected by this model (in particular, entropy of adsorption and self-interaction between 5CB molecules in the liquid state), we suggest that the BE_PhCN_ must be stronger than (more negative than) −0.20 eV for homeotropic anchoring; no interaction occurs for weaker (more positive) BE values, which would correspond to a parallel orientation of the LC relative to the salt substrate surface. We adopt this cutoff value as a model parameter in the absence of an additional set of experiments to rigorously determine the threshold value of BE_PhCN_ required for homeotropic anchoring, which should lie between the smallest computed value for which anchoring is observed (−1.91 eV for Cd^+^) and the largest computed value for which anchoring does not occur (−0.19 eV for Na^0^). We finally note that these threshold values were selected in the context of displacement of 5CB by DMMP. Future experiments may be able to better identify a more precise value of the cutoff energy not only for this system, but also for systems utilizing new liquid crystal-forming molecules with modified functionalities that may have different entropic corrections to the free energy or self-interaction properties than those found in liquid 5CB.

The value of the final charge (after energy minimization of the complex) on the metal atom (*q*_f_) in its interaction with analytes or LCs was obtained through Mulliken population analysis^23^.

### Experimental

Experiments were conducted according to the procedure established in our recent work^24^, which demonstrated that the loading of metal salt deposited on the surface can affect the orientational change of anchored LCs. We note that early studies^11^ used high metal salt loadings that oriented LCs through both LC–cation interactions as well as other intermolecular interactions (for example, through creation of an electrical double layer). The methodology adopted in the present work used metal salt loading that oriented the LCs largely through LC-cation bonds, which is in closer agreement with the adopted computational model.

Metal ions were deposited on the bottom surface of polymeric microwells (200 μm diameter, 5 μm depth) by spincoating 10 mM ethanolic solutions of metal perchlorates at 3000, rpm for 30 s (spincoater from Laurell Technologies, PA). Next, the microwells were filled with 5CB using a micropipette. The microwells were then exposed to a stream of N_2_ containing DMMP (10 p.p.m.) within a flow cell with glass windows, allowing observation of the optical image of 5CB through a polarized optical microscope. The flow of gas containing DMMP was controlled to 300 ml min^−1^ by a rotameter (Aalborg Instruments and Control, Orangeburg, NY). The optical image of the LC was recorded using an Olympus camera (Olympus C2040Zoom, Melville, NY) and WinTV software (Hauppauge, NY). For experiments involving water vapour (that is, no DMMP), microwells were synthesized according to the above procedure and were then exposed to open air in the lab for one hour. The room temperature was 26 °C and the relative humidity was 31% (corresponding to a water vapour concentration around 10,000 p.p.m.).

### Data availability

All final data quantities are contained herein or in the Supplementary Information. Raw data are available from the corresponding author upon request.

## Additional information

**How to cite this article:** Roling, L.T. *et al*. Towards first-principles molecular design of liquid crystal-based chemoresponsive systems. *Nat. Commun.* **7,** 13338 doi: 10.1038/ncomms13338 (2016).

**Publisher's note:** Springer Nature remains neutral with regard to jurisdictional claims in published maps and institutional affiliations.

## Supplementary Material

Supplementary Information Supplementary Figures 1-4, Supplementary Tables 1-5, Supplementary Discussion and Supplementary References.

## Figures and Tables

**Table 1:** Formal charge binding energies and metal charges.

**Me**^**n+**^	**BE**_**PhCN**_	***q***_**f**_	**BE**_**DMMP**_	***q***_**f**_	**BE**_**DMMP**_**–BE**_**PhCN**_	Disp. (Calc)*	**Disp. (Exp)**†	**Agreement**‡
Al^3+^	−24.69	1.03	−19.82	1.74	4.87	No	Yes	No
Fe^3+^	−20.31	1.60	−19.63	1.51	0.68	No	Yes	No
La^3+^	−8.56	2.32	−10.15	2.35	−1.59	Yes	Yes	Yes
Cd^2+^	−6.49	1.22	−7.33	1.39	−0.84	Yes	No	No
Co^2+^	−6.88	0.87	−7.72	1.22	−0.84	Yes	No	No
Cu^2+^	−9.88	0.81	−8.52	1.31	1.36	No	Yes	No
Ni^2+^	−7.66	0.86	−8.26	1.23	−0.60	Yes	No	No
Zn^2+^	−6.81	1.34	−8.17	1.27	−1.36	Yes	No	No
Ag^+^	−2.21	0.73	−2.27	0.79	−0.06	No	No bind	No
Na^+^	−1.41	0.83	−1.71	0.89	−0.30	Yes	No bind	No

Binding energy [eV] and final metal charge (*q*_*f*_) for the minimum energy structures of Me^n+^–PhCN and Me^n+^–DMMP using the formal charge approach. Agreement regarding displacement events between theory and the original experiments was only seen in one of ten cases. After the follow-up experiments, agreement regarding displacement increased to three of ten cases (due to displacement of 5CB from Cd and Zn in the follow-up experiments).

^*^Computed displacement of PhCN by DMMP.

^†^Experimental displacement of PhCN by DMMP, from previous studies^11^.

^‡^Agreement between computed and experimental displacement. ‘No bind' indicates no homeotropic anchoring of the liquid crystal to the metal cation. Displacement is assumed to occur when BE_DMMP_−BE_PhCN_<−0.20 eV.

**Table 2:** Reduced charge binding energies and metal charges.

**Me**^**(n-1)+**^	**BE**_**PhCN**_	***q***_**f**_	**BE**_**DMMP**_	***q***_**f**_	**BE**_**DMMP**_**–BE**_**PhCN**_	Disp. (Calc)*	**Disp. (Exp)**†	**Agreement**‡
Al^2+^	−7.45	1.36	−8.60	1.36	−1.15	Yes	Yes	Yes
Fe^2+^	−5.70	1.58	−8.08	1.37	−2.38	Yes	Yes	Yes
La^2+^	−4.06	1.77	−5.20	1.64	−1.14	Yes	Yes	Yes
Cd^+^	−1.91	0.74	−2.27	0.77	−0.36	Yes	No	No
Co^+^	−2.51	0.83	−2.54	0.61	−0.03	No	No	Yes
Cu^+^	−2.56	0.77	−2.81	0.70	−0.25	Yes	Yes	Yes
Ni^+^	−2.56	0.77	−2.78	0.67	−0.22	Yes	No	No
Zn^+^	−2.23	0.64	−2.79	0.67	−0.56	Yes	No	No
Ag^0^	−0.08	−0.05	−0.17	−0.04	−0.09	No bind	No bind	Yes
Na^0^	−0.19	−0.13	−0.44	−0.14	−0.25	No bind	No bind	Yes

Binding energy [eV] and final metal charge (*q*_*f*_) for the minimum energy structures of Me^n+^–PhCN and Me^n+^–DMMP using the reduced charge approach. Agreement regarding displacement events between theory and experiment was initially seen in seven of ten cases, as shown below. After the follow-up experiments, agreement regarding displacement increased to nine of ten cases (due to displacement of 5CB from Cd and Zn in the follow-up experiments). Further, the disagreement in the Ni case arises from only a very small difference (0.02 eV) between the displacement energy and the adopted energy threshold for the displacement event.

^*^Computed displacement of PhCN by DMMP.

^†^Experimental displacement of PhCN by DMMP, from previous studies^11^.

^‡^Agreement between computed and experimental displacement. ‘No bind' indicates no homeotropic anchoring of the liquid crystal to the metal cation. Displacement is assumed to occur when BE_DMMP_− BE_PhCN_<−0.20 eV.
