# Supplementary material for: Towards first-principles molecular design of liquid crystal-based chemoresponsive systems
Source: Nat Commun. 2016 Nov 2;7:13338. doi: 10.1038/ncomms13338 (PMC5097134; doi:10.1038/ncomms13338)
Supplement: Supplementary Information — Supplementary Figures 1-4, Supplementary Tables 1-5, Supplementary Discussion and Supplementary References. [file ncomms13338-s1.pdf]

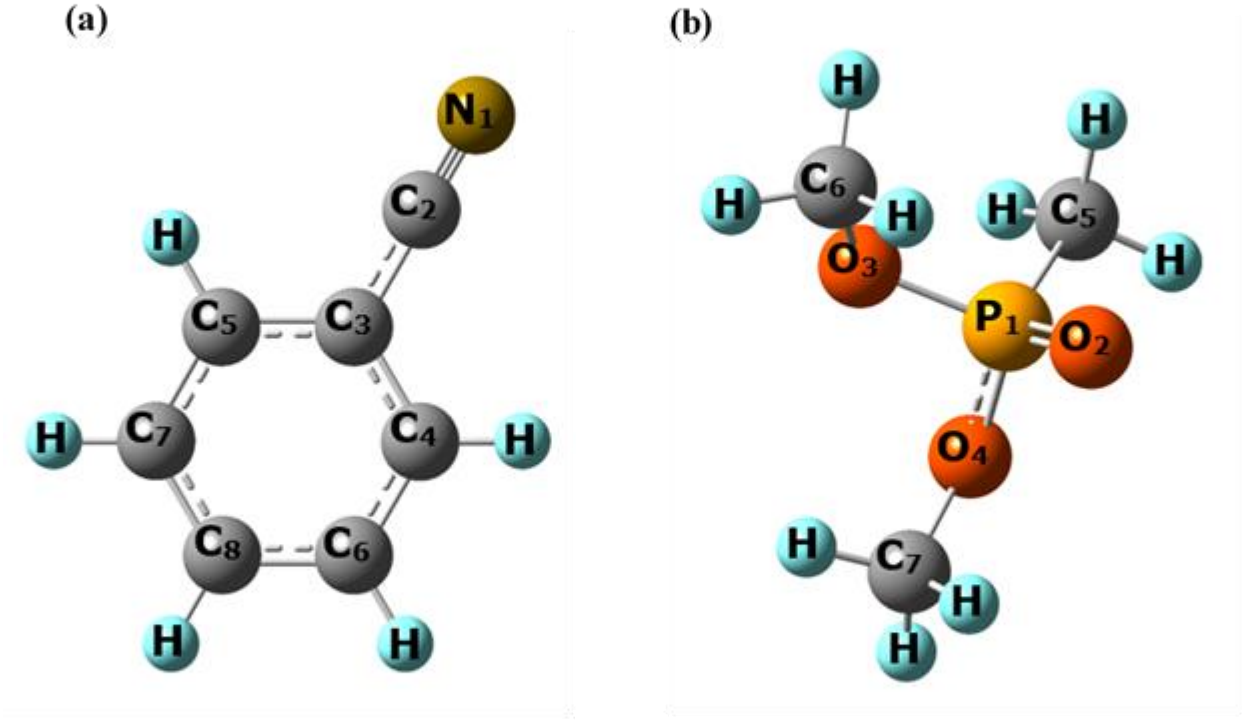

Supplementary Figure 1: Optimized gas-phase structures

Shown are the energy-optimized structures of gas-phase (a) benzonitrile (PhCN) and (b) dimethyl methylphosphonate (DMMP). Optimized structural parameters are provided in Supplementary Table 1 for PhCN and in Supplementary Table 2 for DMMP.

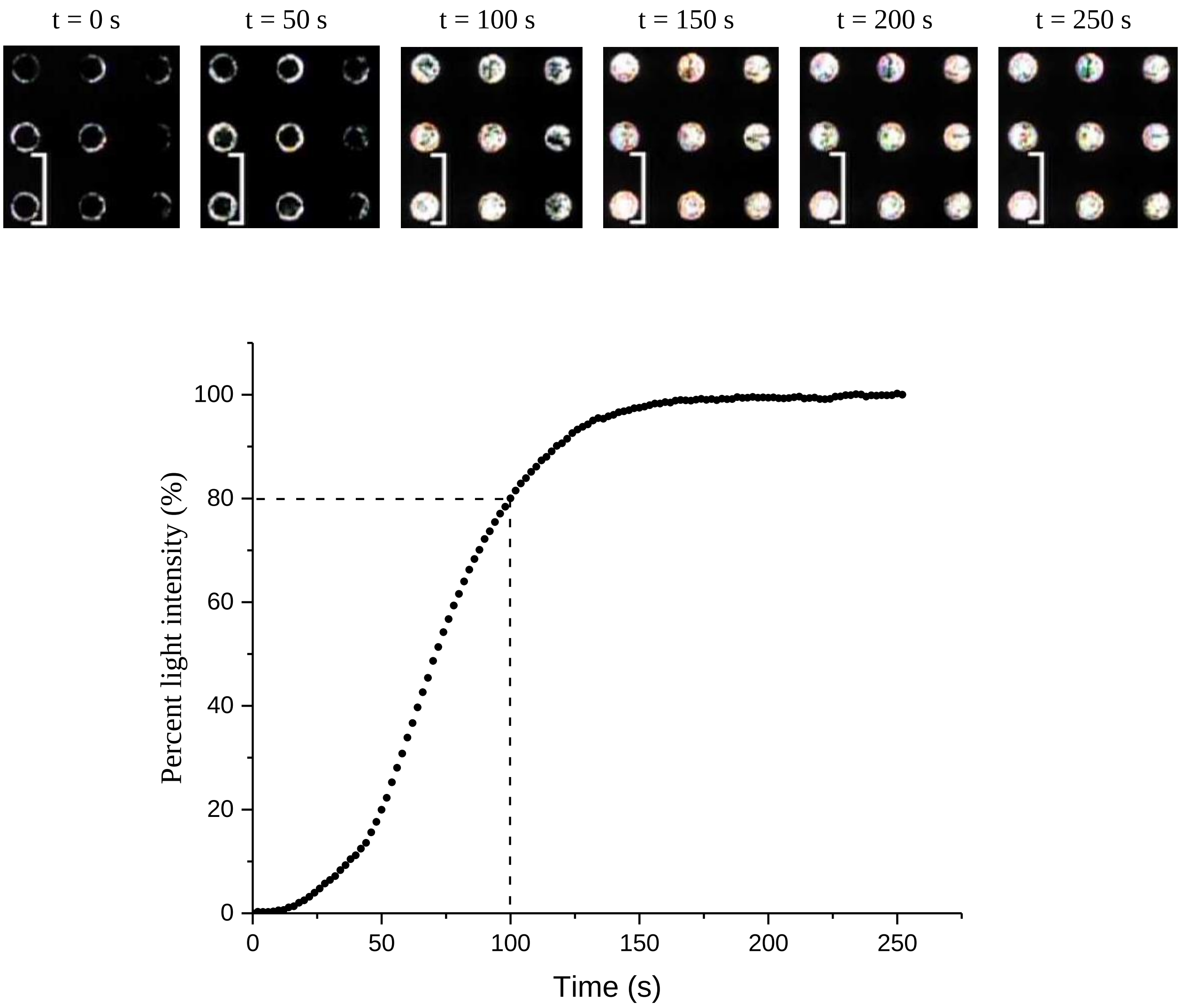

Supplementary Figure 2: Sample dynamic response of 5CB to DMMP exposure

Shown is the response of 5CB anchored to a surface coated with 10 mM Al^{3+} upon exposure to 10 ppm DMMP at t = 0 s. The dotted line indicates the characteristic response time, defined as the time taken to reach 80% normalized light intensity (in this sample, 100 s).

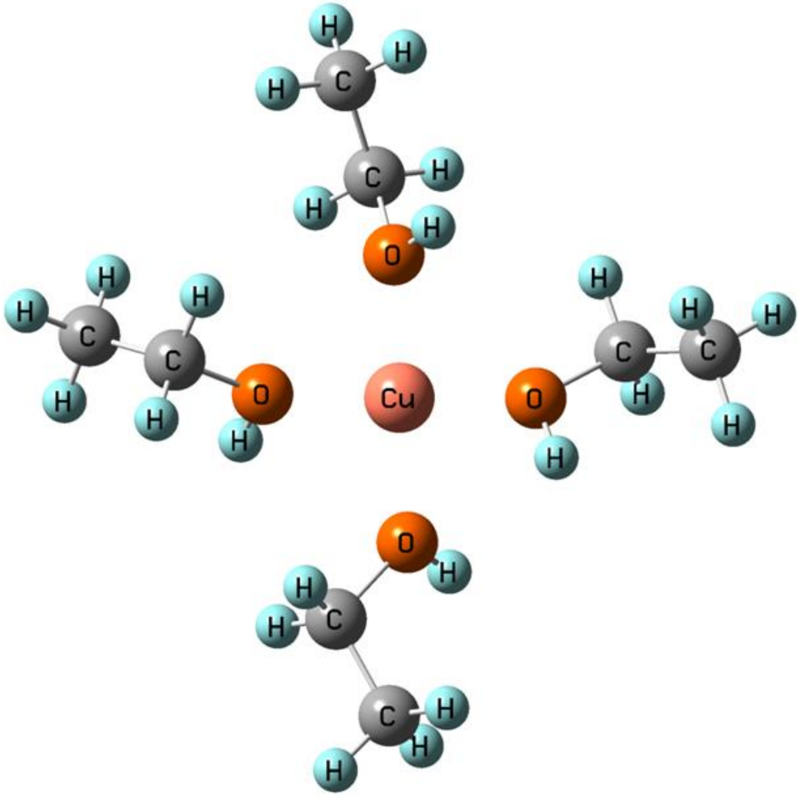

Supplementary Figure 3: Minimum energy structure of solvated Cu^{2+}

Calculated minimum energy structure of Cu^{2+} solvated by four ethanol (EtOH) molecules. Charge density is donated from the solvent to the cation, lowering the effective charge on the cation.

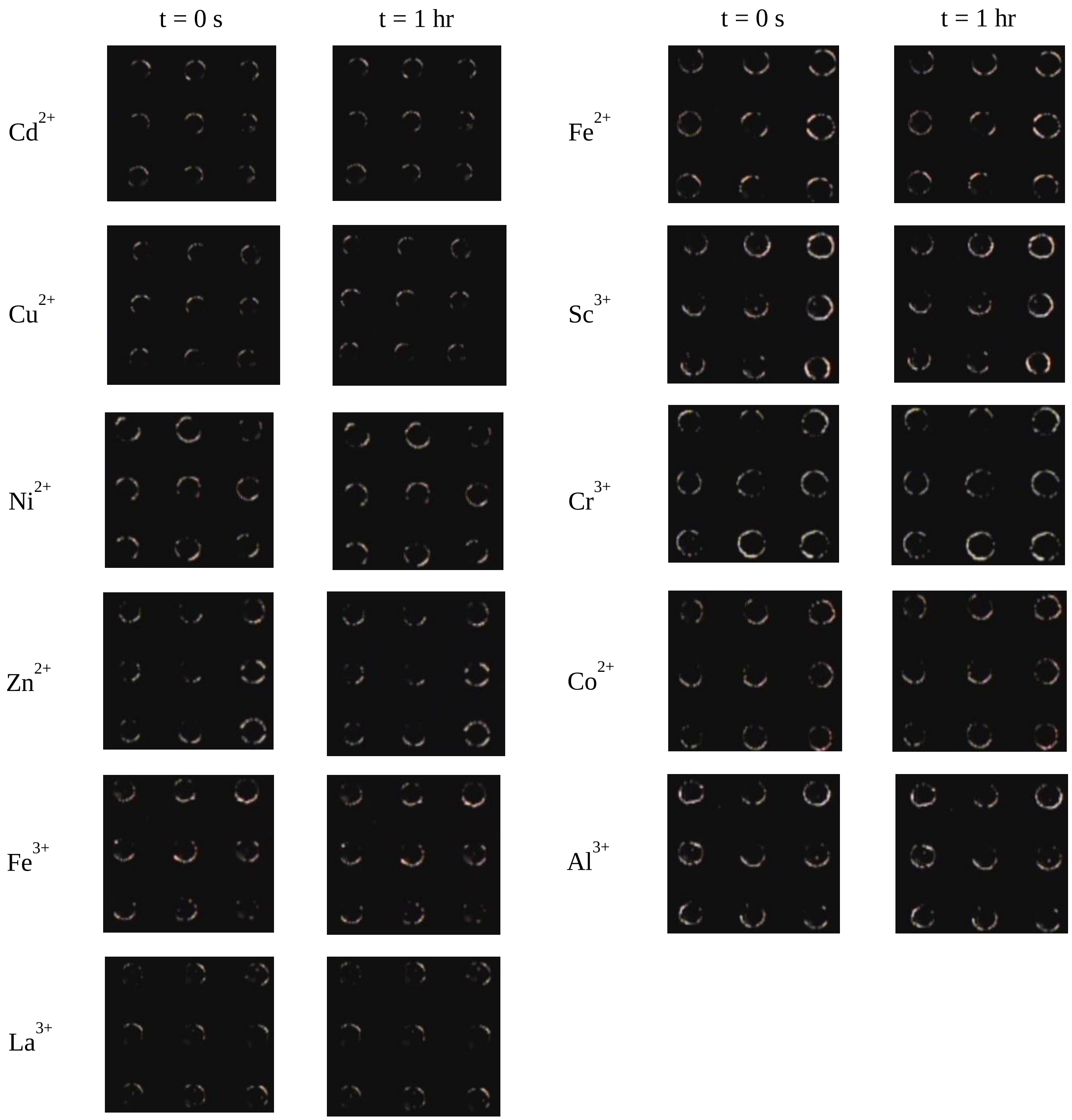

Supplementary Figure 4: Response of 5CB to water exposure

Sample optical response (crossed-polars) of nematic 5CB anchored to a surface coated with 10 mM metal salts upon exposure for one hour to air containing water vapor (temperature was 26 °C and relative humidity was 31%). No change was observed in any sample, indicating that H_2O could not displace 5CB.

Supplementary Table 1: Structural parameters for gas-phase benzonitrile (PhCN)

Main structural parameters [bond lengths in Å, angles in º] for energy-optimized gas phase PhCN. Labels correspond to atoms shown in Supplementary Figure 1a. All C-H bond lengths are between 1.08-1.09 Å.

Parameter	CBS-QB3	B3LYP/CEP-121G	Experimental[1]
$N_1 – C_2$	1.16	1.19	1.16
$C_2 – C_3$	1.43	1.45	1.45
$C_3 – C_4$	1.40	1.42	1.39
$C_4 – C_6$	1.39	1.41	1.40
$C_6 – C_8$	1.39	1.42	1.40
$C_4 – C_3 – C_5$	120.0	120.1	121.6
$C_3 – C_4 – C_6$	119.7	119.7	119.0
$C_4 – C_6 – C_8$	120.2	120.2	120.1
$C_6 – C_8 – C_7$	120.1	120.1	120.1

Supplementary Table 2: Structural parameters for gas-phase dimethyl methylphosphonate (DMMP)

Main structural parameters [bond lengths in Å, angles in º] for energy-optimized gas phase dimethyl methylphosphonate (DMMP). Labels correspond to atoms shown in Supplementary Figure 1b. All C-H bond lengths are between 1.08-1.09 Å.

Parameter	CBS-QB3	B3LYP/CEP-121G	Computed[2]
$P_1 – O_2$	1.47	1.60	1.48 – 1.51
$P_1 – O_3$	1.60	1.74	1.59 – 1.63
$P_1 – O_4$	1.61	1.74	1.61 – 1.65
$P_1 – C_5$	1.80	1.86	1.79 – 1.81
$O_2 – P_1 – C_5$	116.5	121.6	116.4 – 116.8
$O_2 – P_1 – O_3$	117.3	113.4	116.4 – 117.4
$O_2 – P_1 – O_4$	113.4	113.4	113.3 – 113.9
$P_1 – O_3 – C_6$	120.0	120.4	115.5 – 119.7
$P_1 – O_4 – C_7$	121.3	120.4	116.1 – 120.4
$O_3 – P_1 – O_4$	101.3	105.8	100.4 – 101.7

Supplementary Table 3: Structural parameters using the formal charge approach

Selected structural parameters calculated for the energy-optimized Me^{n+} – PhCN and Me^{n+} – DMMP systems using the formal charge approach.

	Me^{n+} – PhCN			Me^{n+} – DMMP		
	Bond Length (Å)		Angle (º)	Bond Length (Å)		Angle (º)
Me^{n+}	N – Me^{n+}	C – N	C – N –Me^{n+}	O – Me^{n+}	P – O	P – O –Me^{n+}
Al^{3+}	2.02	1.16	180.0	1.69	1.64	166.4
Fe^{3+}	1.96	1.16	180.0	1.84	1.55	175.1
La^{3+}	2.23	1.23	180.0	2.08	1.76	166.3
Cd^{2+}	2.14	1.20	147.1	2.12	1.69	103.9
Co^{2+}	1.83	1.15	180.0	1.83	1.56	101.2
Cu^{2+}	1.83	1.16	179.7	1.80	1.55	157.1
Ni^{2+}	1.87	1.15	180.0	1.83	1.56	101.1
Zn^{2+}	1.91	1.17	143.5	1.88	1.55	101.9
Ag^{+}	2.13	1.18	180.0	2.13	1.64	152.4
Na^{+}	2.24	1.16	180.0	2.09	1.50	168.5

Calculated parameters for isolated molecules are 1.16 Å (C – N) and 1.47 Å (P – O).

Supplementary Table 4: Structural parameters using the reduced charge approach

Selected structural parameters calculated for the energy-optimized Me^{n+} – PhCN and Me^{n+} – DMMP systems using the reduced charge approach.

	Me^{n+} – PhCN			Me^{n+} – DMMP		
	Bond Length (Å)		Angle (º)	Bond Length (Å)		Angle (º)
$Me^{(n-1)+}$	N – Me^{n+}	C – N	C – N –Me^{n+}	O – Me^{n+}	P – O	P – O –Me^{n+}
Al^{2+}	2.66	1.15	180.0	1.67	1.61	157.6
Fe^{2+}	1.94	1.16	180.0	1.77	1.58	151.3
La^{2+}	2.39	1.20	180.0	2.18	1.69	172.3
Cd^{+}	2.25	1.19	180.0	2.15	1.64	162.6
Co^{+}	1.83	1.16	180.0	1.86	1.52	147.4
Cu^{+}	1.81	1.16	180.0	1.83	1.51	142.3
Ni^{+}	1.82	1.16	180.0	1.84	1.52	143.4
Zn^{+}	2.02	1.16	180.0	1.93	1.51	157.4
Ag^{0}	2.51	1.19	137.4	2.45	1.62	147.6
Na^{0}	2.37	1.16	147.4	2.23	1.48	172.9

Calculated parameters for isolated molecules are 1.16 Å (C – N) and 1.47 Å (P – O).

Supplementary Table 5: Binding energies of water to metal cations

Comparison of water binding energies to metal cations with the respective binding energies of PhCN and DMMP using the reduced charge approach (all charges listed are reduced charges). The italicized entries correspond to those systems for which homeotropic anchoring is not observed experimentally. All values are in eV.

Cation	BE(H_2O)	BE(PhCN)	BE(H_2O) - BE(PhCN)	BE(DMMP)	BE(H_2O) - BE(DMMP)
Al^{2+}	-4.25	-7.45	3.20	-8.60	4.35
Cr^{2+}	-3.14	-5.62	2.48	-7.17	4.03
Fe^{2+}	-3.57	-5.70	2.13	-8.08	4.51
La^{2+}	-2.66	-4.06	1.40	-5.19	2.53
Sc^{2+}	-2.63	-4.09	1.46	-5.47	2.84
Cd^{+}	-1.44	-1.91	0.47	-2.27	0.83
Co^{+}	-1.53	-2.51	0.98	-2.54	1.01
Cu^{+}	-1.62	-2.56	0.94	-2.81	1.19
Fe^{+}	-1.33	-2.27	0.94	-3.00	1.67
Ni^{+}	-1.67	-2.56	0.89	-2.78	1.11
Zn^{+}	-1.44	-2.23	0.79	-2.79	1.35
Ag^{0}	*-0.13*	*-0.08*	*-0.05*	*-0.17*	*0.04*
Na^{0}	*-0.21*	*-0.19*	*-0.02*	*-0.44*	*0.22*

Supplementary Discussion

Energy Minimization of DMMP and PhCN

Supplementary Figure 1 shows the energy-optimized structure of the individual gas-phase PhCN and DMMP molecules, as determined by our electronic structure calculations. Supplementary Table 1 summarizes the main structural parameters of PhCN, along with the respective experimental values[1]. The structural parameters for DMMP are shown in Supplementary Table 2 alongside previously computed values[2] at different levels of theory; to the best of our knowledge, no experimental values for DMMP structural parameters are available. All C – H bond lengths are between 1.08 and 1.09 Å for both molecules. In general, the parameters computed at the CBS-QB3 level of theory agree with the experimental or previously computed parameters better than those at the B3LYP/CEP-121G level of theory.

Energy Minimization of DMMP and PhCN Complexes with Metal Cations

Supplementary Table 3 shows the main structural parameters of the Me^{n+}-PhCN and Me^{n+}-DMMP complexes with the formal charge approach, and Supplementary Table 4 shows the main structural parameters of those complexes with the reduced charge approach. The interaction of metal cations with the PhCN molecule only slightly affects the C–N bond length (1.16 Å with CBS-QB3 for isolated PhCN), with the exception of PhCN–La^{3+} (1.23 Å), while the interaction between Me^{n+} and DMMP generally leads to an increased P–O bond length (1.47 Å for isolated DMMP). The C–N–Me^{n+} bond angle is 180° (i.e. linear), except for the cases of Zn^{2+}, Cd^{2+}, Ag^0 and Na^0, in which slight bending occurs. No particular trend is observed for the P–O –Me^{n+} bond angle. The N–Me^{n+} bond length is generally longer than the O–Me^{n+} bond length.

Correlation of Response Time with Calculated Binding Energies

An exponential curve relating the experimental response time to the calculated binding energies was chosen for fundamental reasons underlying the displacement of the liquid crystal from the surface. In particular, we can understand the experimental response time in the context of reaction rates; specifically, the inverse of the response time corresponds to the reaction rate. Assuming an Arrhenius-type expression for reaction rates, we can write:

$$\frac{1}{response\ time} \propto rate = (\prod_i [C_i]) * A * \exp(\frac{-E_A}{RT}) \quad (1)$$

where $[C_i]$ are the respective concentration terms, A is the pre-exponential term, E_A is the activation energy, R is the gas constant, and T is the absolute temperature. In the context of this displacement reaction, we assume that $[C_i]$ and A are invariant when changing metal cations, so the response time is determined directly by the exponential term. Assuming that there exists a Brønsted-Evans-Polanyi correlation[3,4] between the activation energy of the displacement event (E_A) and the reaction free energy (ΔG), this allows approximation of activation energies as a linear function of the reaction energy; i.e., $E_A = \alpha * (\Delta G) + \beta$, for some parameters α and β. Since ΔG for this displacement is dominated by the difference in binding energies (i.e. $BE_{\mathrm{DMMP}} - BE_{\mathrm{PhCN}}$) due to consistent entropic effects, E_A can be replaced in the exponential by the difference in binding energies. The parameter β can be grouped into a modified pre-exponential term along with the constant concentration terms, and the entire expression inverted to yield the final expressions for response time as given in our model.

$$\frac{1}{response\ time} \propto (\prod_i [C_i]) * A * \exp(\frac{-\{\alpha(BE_{DMMP} - BE_{PhCN}) + \beta\}}{RT}) \quad (2)$$

$$response\ time \propto A' * \exp(\frac{\alpha(BE_{DMMP} - BE_{PhCN})}{RT}) \quad (3)$$

In this sense, our exponential fit provides us the values of α and A' corresponding to this reaction event. We note that exact determination of the terms contained in A' would require experiments outside the scope of this work. The exact response time would clearly depend on the value of the exponential and all the parameters lumped into A'.

Charge Transfer from Ethanol Solvent

We hypothesize that some residual ethanol (EtOH) molecules from experimental preparation of the substrate surface containing the metal cations might remain bound to the metal cation as a possible consequence of a strong interaction between the cation and EtOH in solution, and that the charge of the solvated metal cation is therefore different from its formal charge due to donation of electron density from the EtOH solvent to the cation. To test this hypothesis, we calculated the differential binding energy (BE_{diff}) and optimized cation charge (q_f) resulting from the relaxation of a Cu^{2+} cation (formal charge) in the presence of n EtOH molecules (n = 1 – 4). BE_{diff} for the nth EtOH molecule is defined as follows:

$$BE_{\text{diff}} = E_{\text{Me+}n\text{EtOH}} - E_{\text{Me+}(n\text{-}1)\text{EtOH}} - E_{\text{EtOH}} \qquad (4)$$

where $E_{\text{Me+}n\text{EtOH}}$ and $E_{\text{Me+}(n\text{-}1)\text{EtOH}}$ are the total energies of the complex with n and (n-1) EtOH molecules, respectively, and E_{EtOH} is the total energy of the isolated EtOH molecule.

The optimized structure of $[Cu(EtOH)_4]^{2+}$ is shown in Supplementary Figure 6. All O – Cu distances in $[Cu(EtOH)_4]^{2+}$ are calculated to be between 1.95-1.96 Å, which agree with the experimental value of 1.97 Å reported by Inada et al. for the solvation of Cu(II) by an average of four EtOH molecules[5]. We find that q_f decreases to 1.47, 0.68, 0.59, 0.31 as n increases from 1 to 4, respectively. These data suggest that the solvated Cu cation has a q_f smaller than (2+) and its value is closer to the charge used in the reduced charge approach, particularly when the Cu cation

interacts with 1-2 EtOH molecules (q_f is a strong function of n). BE_{diff} also decreases to -6.1 eV, -4.6 eV, -2.2 eV, -1.9 eV as n increases from 1 to 4, respectively, which qualitatively follows our observation that cations in the reduced charge model bind species more weakly than those in the formal charge model. The decrease of the metal charge as function of the number of EtOH molecules in the complex is in agreement with related computational studies of metal cation solvation by Rao et al. [6] and by Xu et al. [7].

In order to further investigate this phenomenon, we computed BE_{PhCN} and BE_{DMMP} to Cu^{2+} in the presence of one and two EtOH molecules. In the presence of one EtOH molecule, we calculated BE_{PhCN} = -6.08 eV and BE_{DMMP} = -4.86 eV. In this case, the BEs are between the values obtained using the reduced charge approach (-2.56, -2.81) and those obtained with the formal charge approach (-9.88, -8.52). Importantly, since PhCN binds more strongly than DMMP in this calculation, the model predicts that displacement of PhCN by DMMP will not occur, in contrast to experimental results. We performed the same calculations in the presence of two EtOH molecules, obtaining BE_{PhCN} = -2.99 eV and BE_{DMMP} = -3.73 eV, which would predict displacement of PhCN by DMMP, in agreement with experiments. These two values are within 1 eV of the values calculated with the reduced charge approach, demonstrating reasonable qualitative agreement between this larger system containing Cu^{2+} bound to two EtOH molecules and the reduced charge model.

Effect of Charge Donation from Chemical Species in the Sensing Surface Environment

In addition to the ethanol solvent, other species may contribute to the donation of charge density to metal cations, thereby reducing their charge from the formal charge in the metal salt precursor. As a brief example, water is omnipresent in the environment of our experiments and

can also donate charge through coordination with the metal cation through one of its lone electron pairs. However, we calculate that a metal cation interacts with water substantially more weakly than with ethanol. For example, a single water molecule binds to Cu^{2+} with an energy of -4.56 eV, which is weaker than the binding of both the first (-6.1 eV) and second (-4.6 eV) EtOH molecules described earlier. Further, the binding energy of water to Cu^{1+} (reduced charge) is just -1.60 eV, so once Cu^{2+} is reduced in the presence of two EtOH molecules, adsorption of a third and fourth EtOH molecule would still be preferable to adsorption of water. The metal cation will therefore strongly prefer to maintain coordination to any trace EtOH remaining from synthesis of the salt surface rather than coordinate to free water molecules, justifying our selection of EtOH as the charge-donating species in our model.

Supplementary References

1 Hellwege, K. H. H. A. M. *Landolt-Bornstein: Group II: Atomic and Molecular Physics Volume 7: Structure Data of Free Polyatomic Molecules*. (Springer-Verlag, 1976).

2 Yang, L., Shroll, R. M., Zhang, J. X., Lourderaj, U. & Hase, W. L. Theoretical Investigation of Mechanisms for the Gas-Phase Unimolecular Decomposition of DMMP. *J. Phys. Chem. A* **113**, 13762-13771 (2009).

3 Evans, M. G. & Polanyi, M. Inertia and driving force of chemical reactions. *Trans. Faraday Soc.* **34**, 11-24 (1938).

4 Bell, R. P. The Theory of Reactions Involving Proton Transfers. *Proc. R. Soc. Lond. A* **154**, 414-429 (1936).

5 Inada, Y., Hayashi, H., Sugimoto, K. & Funahashi, S. Solvation structures of Manganese(II), iron(II), cobalt(II), nickel(II), copper(II), zinc(II), and gallium(III) ions in methanol, ethanol, dimethyl sulfoxide, and trimethyl phosphate as studied by EXAFS and electronic spectroscopies. *J. Phys. Chem. A* **103**, 1401-1406 (1999).

6 Rao, J. S., Dinadayalane, T. C., Leszczynski, J. & Sastry, G. N. Comprehensive Study on the Solvation of Mono- and Divalent Metal Cations: Li+, Na+, K+ Be2+, Mg2+ and Ca2+. *J. Phys. Chem. A* **112**, 12944-12953 (2008).

7 Xu, M. J., Dou, X. M., Bu, Y. X. & Zhang, Y. F. Density functional theory calculations for the microsolvation of M3+-zwitterionic glycine complexes (M3+ = Al3+, Ga3+, In3+). *Chem. Phys. Lett.* **537**, 101-106 (2012).
